# Compassion Fatigue and Perceived Organizational Performance: Emergency Nurses’ Wellbeing as a Mediating Factor

**DOI:** 10.1155/jonm/9669754

**Published:** 2026-11-03

**Authors:** Sally Mohammed Farghaly Abdelaliem, Haitham Mokhtar Mohamed Abdallah, Mohamed Ezzelregal Abdelgawad

**Affiliations:** ^1^ Department of Nursing Faculty of Allied Health Sciences Kuwait University Kuwait City Kuwait kuniv.edu; ^2^ Nursing Administration Department Faculty of Nursing Alexandria University Alexandria Egypt alexu.edu.eg; ^3^ Medical-Surgical Nursing Department College of Nursing Jouf University Sakaka Saudi Arabia ju.edu.sa; ^4^ Critical Care and Emergency Nursing Department Faculty of Nursing Alexandria University Alexandria Egypt alexu.edu.eg

**Keywords:** compassion fatigue, cross-sectional studies, mediation analysis, nurses, nurse managers, nurse wellbeing, perceived organizational performance, secondary traumatic stress

## Abstract

**Background:**

Compassion fatigue is a widespread professional issue in nursing practice, yet the wider organizational implications of compassion fatigue, especially its association with perceived organizational performance through nurses’ wellbeing, are not fully investigated in the context of Egyptian critical care. Clarifying this pathway is important for designing targeted managerial interventions that can maintain the wellbeing of the staff and the efficiency of the institution.

**Aim:**

To investigate the mediating effect of nurses’ wellbeing on the relationship between compassion fatigue and perceived organizational performance among critical care and emergency nurses.

**Method:**

A descriptive correlational cross‐sectional study was undertaken with 111 nurses who worked in intensive care (54.1%), emergency (13.5%), and other inpatient and outpatient units of Alexandria Main University Hospital, Egypt. Three validated data collection instruments were used to collect data between July and September 2024, namely, the Professional Quality of Life (ProQOL) Secondary Traumatic Stress (STS) subscale, used to measure compassion fatigue (10 items, 5‐point Likert scale; *α* = 0.868); the Organizational Performance Questionnaire, which captures perceived organizational performance (11 items, 5‐point Likert scale; *α* = 0.944); and the Nurse Wellbeing Scale (17 items, 10‐point Likert‐type scale; *α* = 0.945). The tools were subject to intensive translation, content validation, exploratory and confirmatory factor analysis (AMOS 23.0), and reliability testing. Associations were examined with Pearson correlation and regression‐based mediation analysis (PROCESS Model 4; 5000 bootstrap samples).

**Results:**

Most nurses (65.8%) expressed a low degree of compassion fatigue (mean = 2.70 ± 0.74), and 44.1% perceived organizational performance as low (mean = 3.04 ± 0.84). Nurses’ wellbeing was low (mean = 4.02 ± 1.98 on the 1–10 scale), with the largest gaps in physical self‐care interventions, such as exercise, sleep, or mindfulness, and lack of manager–nurse communication about wellbeing. Compassion fatigue had a significant negative correlation with perceived organizational performance (*r* = −0.400, *p* < 0.001) and wellbeing (*r* = −0.513, *p* < 0.001). In the mediation analysis, higher compassion fatigue was significantly associated with lower wellbeing (*B* = −2.329, *p* < 0.001), and that wellbeing was a positive predictor of perceived organizational performance (*B* = 0.154, *p* < 0.001). After accounting for wellbeing, the direct effect of compassion fatigue on perceived organizational performance was nonsignificant (*B* = −0.140, 95% CI [‐0.356, 0.077], *p* = 0.196), whereas the indirect effect through wellbeing was significant (*B* = −0.360, 95% bootstrap CI [−0.545, −0.227]), indicating that wellbeing statistically mediated 72.0% of the total effect.

**Conclusion:**

Nurses’ wellbeing statistically mediated the association between compassion fatigue and perceived organizational performance. Because of the cross‐sectional design, these findings indicate associations rather than causal effects, and longitudinal studies are needed to confirm the pathway.

**Implications for Nursing Management:**

Systematic, frequent screening for compassion fatigue using instruments such as the ProQOL is recommended, together with organizational‐level interventions that address self‐care deficits such as protected scheduling and resources dedicated to wellbeing. Supporting leadership, creating psychologically safe workplaces, implementing formal peer support networks, and infusing compassion fatigue prevention into education are recommended to support nurse wellbeing and perceived organizational performance, although these recommendations require testing in longitudinal and interventional studies.

## 1. Introduction

The cost of nursing workforce loss, clinical or medical errors, and deteriorating quality of care has led healthcare organizations across the globe to take a closer look at the psychological and occupational experiences of their nursing employees [1]. Of the professional risks that nurses have to deal with, compassion fatigue is one of the most widespread ones and its impact on a nurse can be deeply experienced and felt even by other professionals [2]. Compassion fatigue refers to the emotional and physical exhaustion of the nurse due to protracted emotional involvement with patients in a path that is gradually depleting the nurse of their compassionate care and, as the evidence continues to mount, weakening the institutions within which they practice [3].

Emergency and critical care nurses are especially susceptible. They operate in settings that have high patient acuity, tight decision‐making schedules, traumatic experiences, and night shift schedules that do not allow much time to relax [4]. These circumstances hasten the compassion fatigue trajectory and, at the same time, lower the same resources emotional energy, cognitive availability, and interpersonal engagement needed by the nurses to continue contributing to the organization [5]. However, the particular organizational implications of compassion fatigue and how those implications are generated have not been sufficiently theorized within the context of Egyptian public hospitals.

Perceived organizational performance is an aspect of nursing that indicates the extent to which an institution meets its operational, quality, and developmental objectives using its nursing workforce [6]. Depletion causes nurses to disengage themselves with organizational systems, withdraw themselves in the quality improvement activities and lessen the discretionary effort that provides cohesion and patient safety to an institution. Whether compassion fatigue hurts perceived organizational performance or not, the connection between these two variables is widely understood, but what is the pathway of its occurrence, and what lies in between the two variables.

The wellbeing of nurses is a good nominee of such an intermediate position. Wellbeing involves physical health care, psychological stability, career satisfaction, capacity to cope with work stress, and involvement in self‐care activities [7]. In conditions of good health, nurses have the resource reserves to take up the occupational stress without disengaging from the organization. Whether wellbeing helps to explain the link between compassion fatigue and perceived organizational performance among Egyptian intensive care unit (ICU) and emergency nurses has not been tested empirically and is examined in the present study [8].

Although this argument is considered theoretically plausible and practically urgent, no study has tested the mediation of the wellbeing of nurses in the compassion fatigue‐perceived organizational performance relationship by path analysis in an Egyptian acute care setting. This paper bridges that divide.

### 1.1. Significance of the Study and Research Gap

The phenomenon of compassion fatigue is now well‐documented in nursing specialties, although the majority of studies have focused on its effects at the personal‐level burnout, turnover intention, and job dissatisfaction instead of the organizational level [3, 9]. The organizational effects of compassion fatigue, and the institutional circumstances mediating or moderating such effects, are understudied, especially in low‐and‐middle‐income healthcare systems, where resources are continually constrained [10].

The Egyptian system of state‐provided hospitals is particularly vulnerable to the pressures of the nursing workforce: high patient‐to‐nurse ratios, uneven shift patterns, inadequate occupational health facilities, and a high proportion of young and early‐career nurses in the critical care units [11]. Such circumstances form precisely the atmosphere where compassion fatigue thrives, and deficits of wellbeing are amassed. But Egyptian nursing studies have seldom explored such dynamics with the analytical detail, mediation analysis, and path modeling required to produce actionable management information.

The idea of nurses’ wellbeing has gained significant traction in international nursing policy over the past years, partly due to the acceleration of occupational distress caused by the COVID‐19 pandemic [7, 12]. Although wellbeing has been defined as an organizational resource that acts as a buffer against occupational stressors, its mediating effect between compassion fatigue and organizational outcomes has not been empirically measured with validated Egyptian‐adapted measures and bootstrapping analysis. The gap that our study will fill is that it will present evidence that can be directly applied to nursing management practice within the Egyptian hospital setting and similar areas in the region.

### 1.2. Theoretical Framework and Hypothesis Formulation

The paper will be based on the Conservation of Resources (COR) theory [13] that suggests that people expend energy to gain, develop, and maintain resources in the form of personal energy, emotional capacity, social support, and occupational stability. Psychological stress occurs when such resources are put at risk or lost. Compassion fatigue is a long‐term drain on resources in the nursing setting: The resources of empathy, cognitive availability, and emotional stability of the nurse are gradually drained by patients suffering, which the nurse cannot avoid being exposed to. This burnout directly compromises the wellbeing of nurses, their ability to preserve physical health, deal with occupational stress, and continue to be professionally satisfied. Weakened wellbeing, in its turn, decreases the organizational involvement and input of nurses into the institutional performance objectives.

One secondary model is the Job Demands–Resources (JD–R) model [14] that differentiates between job demands (elements that demand sustained effort, which come at a physical and psychological cost) and job resources (elements that allow goal achievement and mitigate the effects of demand). Compassion fatigue is essentially an increased job demand that on the one hand drains personal resources (wellbeing) and on the other hand lowers the ability of the nurse to take advantage of the job resources available. This logic is operationalized in the mediation model that we test in our study: Compassion fatigue exhausts wellbeing, and the exhaustion of wellbeing is then converted to the performance of an organization. The conceptual framework is suggested in Figure 1.

**FIGURE 1:**
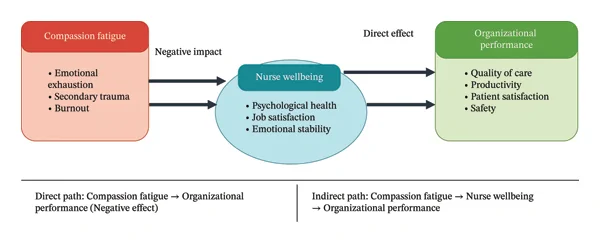
Conceptual framework of the study variables.

### 1.3. Study Aim

The study aims to investigate the mediating effect of nurses’ wellbeing on the relationship between compassion fatigue and perceived organizational performance among critical care and emergency nurses.

### 1.4. Research Questions

i. What are the compassion fatigue levels among critical care and emergency nurses?
ii. What are the levels of perceived organizational performance perceived by nurses?
iii. What is the wellbeing level of nurses and what are the areas of wellbeing that indicate the greatest shortcomings?
iv. Do compassion fatigue, nurses’ wellbeing, and perceived organizational performance have a significant relationship?
v. Is the wellbeing of nurses a significant mediator between compassion fatigue and perceived organizational performance?

### 1.5. Study Hypotheses

 H1. Compassion fatigue and perceived organizational performance have a negative relationship of significant nature in critical care and emergency nurses.
 H2. Compassion fatigue and wellbeing of nurses have a strong negative correlation.
 H3. Wellbeing of nurses is significantly related to the perceived organizational performance in a positive way.
 H4. The mediating role of nurses’ wellbeing plays an important role in the relationship between compassion fatigue and perceived organizational performance.

## 2. Methods

### 2.1. Design

A cross‐sectional descriptive correlational research design was used. This design enabled simultaneous measurement of compassion fatigue, nurses’ wellbeing, and perceived organizational performance across the sample at a single point in time, and is appropriate for examining mediated relationships when time constraints prevent longitudinal data collection [15]. Path analysis with bootstrapping was applied to test the mediation model.

### 2.2. Study Setting

The research was carried out in Alexandria Main University Hospital (AMUH), the biggest state‐owned tertiary referral hospital in Alexandria, Egypt. AMUH is attached to the Faculty of Nursing in Alexandria University, and it provides a high‐volume and complex patient population comprising emergency, critical care, and specialty patients. The involved units were the ICU, emergency room (ER), dialysis unit, cardiac care, specialty critical care unit, and outpatient clinics. These environments reflect the entire continuum of high‐acuity nursing care environments in Egyptian public hospitals and can be defined by the prolonged exposure to patients and the organizational demands that predispose compassion fatigue research to be specifically applicable.

### 2.3. Study Participants and Sampling

The target population was all registered nurses working in the critical care and emergency department of AMUH. A convenience sampling method was employed, where all nurses in all the participating units and all three shifts were recruited in order to have representation with diverse backgrounds in terms of specialty, experience, and conditions of the shift. The criteria were active work as a registered nurse in a participating unit and the willingness to participate and provide informed consent. Nurses who worked purely in administration were not included.

Estimation of the sample size was done using G^∗^ Power 3.1 with the following settings: effect size (*f* ^2^ = 0.15, *α* = 0.05), statistical power (1−β) = 0.80, and a maximum of three predictors in the mediation model, which gave a minimum sample of 77 participants. A total of 145 questionnaires were distributed to ensure that the expected attrition and unfinished surveys were taken into consideration. Of these, 111 questionnaires were returned complete and eligible for analysis, yielding a response rate of 76.6% (111/145).

### 2.4. Study Measurements

A researcher‐developed sociodemographic and work‐related form captured participants’ age, gender, marital status, working unit, educational qualification, years of general nursing experience, and years of experience in the current unit.

### 2.5. Compassion Fatigue (Professional Quality of Life Scale [ProQOL] Secondary Traumatic Stress [STS] Subscale)

Compassion fatigue was operationalized using the 10‐item STS subscale of the ProQOL developed by Stamm [16]. In the ProQOL framework, compassion fatigue comprises two components, burnout and STS; the present study measured only the STS component, which reflects fear‐ and trauma‐related distress arising from exposure to patients’ suffering (e.g., intrusive thoughts, avoidance, feeling on edge). The ProQOL burnout and compassion satisfaction subscales were not administered. Therefore, “compassion fatigue” in this paper refers to STS‐based compassion fatigue, and the findings should not be generalized to the burnout component or to the full construct. Responses are scored on a five‐point Likert scale (1 = *never* to 5 = *very often*), with higher scores indicating greater compassion fatigue. The scale has demonstrated strong cross‐cultural validity and reliability across nursing populations [16]. In this study, it achieved a Cronbach’s *α* of 0.868, reflecting good internal consistency. Levels were classified using the percentage of the possible score range, calculated as (mean score − 1)/4 × 100: low (< 50%, i.e., mean < 3.0), moderate (50% to < 75%, i.e., mean 3.0 to < 4.0), and high (≥ 75%, i.e., mean ≥ 4.0). These are study‐specific cutoffs and differ from the raw‐score cutoffs in the ProQOL manual.

### 2.6. Perceived Organizational Performance Questionnaire

Perceived organizational performance was assessed using the 11‐item questionnaire developed by Milky [6], an unpublished Master’s thesis instrument, capturing nurses’ perceptions of organizational effectiveness across communication, policies, developmental opportunities, and performance appraisal systems. Written permission to use and adapt the tool was obtained from the developer. Items are scored on a five‐point Likert scale (1 = *never satisfied* to 5 = *highly satisfied*), with higher scores reflecting more positive perceived organizational performance. In this study, the scale achieved a Cronbach’s *α* of 0.944, confirming excellent reliability. Mean scores below 3.0 were classified as unsatisfied and at or above 3.0 as satisfied. Because this tool captures nurses’ self‐reported perceptions rather than objective institutional performance indicators, the outcome is referred to as perceived organizational performance throughout this paper.

### 2.7. Nurses’ Wellbeing Scale

The measure of wellbeing in nurses was based on the 17‐item Nurse Wellbeing Scale created by Keener [7], an unpublished doctoral dissertation instrument for which written permission to use and adapt was obtained from the developer, and it evaluates professional fulfillment, self‐care practices, physical health behaviors, psychological resilience, peer wellbeing impact, and organizational communication about wellbeing. The items are rated on a 10‐point Likert‐type scale (1 = *strongly disagree*/*never* to 10 = *strongly agree*/*always*), where the higher the score, the higher the wellbeing. Four items (Items 2, 3, 4, and 5) are negatively worded, reflecting coworker stress, coworker burnout, COVID‐19 impact, and work‐related strain on personal life. Consistent with the developer’s scoring protocol, these items were reverse‐scored (transformed as 11 minus the raw item response) before computing the item‐ and scale‐level means reported in Table 1 and the wellbeing composite used in the correlation, regression, and mediation analyses, as well as for the reliability (Cronbach’s *α*) and confirmatory factor analysis procedures. Accordingly, higher scores on all 17 items, including Items 2, 3, 4, and 5, consistently indicate higher wellbeing. Item‐level means for Items 2–5 in Table 1 are reported after reverse scoring. The scale had a Cronbach’s *α* of 0.945 in this research, which reflects high internal consistency.

**TABLE 1:** Participants’ levels of perception regarding nurses’ wellbeing (*n* = 111).

Items	Mean	±	SD	Low no.	Low %	Moderate no.	Moderate %	High no. (%)
1. The work I do is meaningful to me.	5.07	±	3.14	68	61.3	13	11.7	30 (27.0%)
2. My coworkers demonstrate signs of work‐related stress and anxiety.	4.05	±	2.50	87	78.4	10	9.0	14 (12.6%)
3. My coworkers demonstrate signs of work‐related burnout.	4.98	±	2.94	74	66.7	7	6.3	30 (27.0%)
4. COVID‐19 has negatively impacted my overall wellbeing.	3.91	±	2.71	84	75.7	11	9.9	16 (14.4%)
5. Work‐related stress and burnout negatively impact my personal life.	4.69	±	3.02	75	67.6	10	9.0	26 (23.4%)
6. My coworkers’ wellbeing impacts my personal wellbeing.	4.23	±	2.63	80	72.1	12	10.8	19 (17.1%)
7. I am able to manage work‐related stress and anxiety.	4.62	±	2.61	74	66.7	13	11.7	24 (21.6%)
8. I routinely practice self‐care.	4.69	±	2.77	73	65.8	13	11.7	25 (22.5%)
9. I have a healthy diet.	3.82	±	2.81	80	72.1	15	13.5	16 (14.4%)
10. I am able to decompress after work.	4.25	±	2.68	78	70.3	12	10.8	21 (18.9%)
11. I am able to manage feelings of burnout.	4.36	±	2.68	74	66.7	18	16.2	19 (17.1%)
12. I exercise regularly.	2.77	±	2.47	97	87.4	4	3.6	10 (9.0%)
13. I have healthy sleep habits.	2.89	±	2.48	95	85.6	6	5.4	10 (9.0%)
14. I am comfortable discussing my wellbeing with my manager (s).	3.14	±	2.58	89	80.2	13	11.7	9 (8.1%)
15. I regularly engage in mindfulness practices.	2.96	±	2.51	93	83.8	9	8.1	9 (8.1%)
16. Your fulfillment in your career as a nurse.	4.55	±	2.67	73	65.8	15	13.5	23 (20.7%)
17. Your personal wellbeing.	3.41	±	2.69	89	80.2	8	7.2	14 (12.6%)
Overall nurses’ wellbeing	4.02	±	1.98	84	75.7%	22	19.8%	5 (4.5%)

*Note:* Item scores: Low, 1–5. Moderate, 6–7. High = 8–10 (scale range 1–10). Items 2–5 are shown after reverse scoring (11 − raw score), so higher values indicate higher wellbeing on all items. Overall wellbeing: Low = < 50% of the possible score range. Moderate = 50% to < 75%. High = ≥ 75% (percentage = (sum − 17)/153 × 100).

### 2.8. Study Tools Adaptation, Validity, and Reliability

#### 2.8.1. Tools Translation

To help all three instruments fit the Egyptian cultural and healthcare context, provide the semantic precision, and remove the dangers to construct validity, all three instruments were translated into the Arabic language with the use of the back‐to‐back method of translation [17]. The initial translation was done by a bilingual nursing scholar; the back‐translation was done by an independent bilingual professional. Any discrepancies in the original and back‐translated version were sorted out by consensus review of both translators and the principal investigator.

#### 2.8.2. Content Validity

Content validity was reviewed by a panel of seven academics including three professors of nursing administration and four professors of critical care and emergency nursing. Each instrument was evaluated by the panel in terms of item relevance, clarity, representativeness, and cultural appropriateness in the Egyptian healthcare setting. There were some word‐choice and phrasing changes that were made in reaction to panel feedback. A pilot study was then conducted using 20 nurses sampled in the study setting (not in the final sample) to determine the clarity, applicability, and average time taken to complete the questionnaires. No additional adjustments were necessary after the pilot, and this meant that the average time of completion was about 15–20 min.

#### 2.8.3. Construct Validity

To determine construct validity of the translated tools, both exploratory factor analysis (EFA) and confirmatory factor analysis (CFA) were used. The EFA was normalized with Kaiser normalization using Promax rotation, with the aim of establishing the dimensions of underlying factors that explained variance in the responses to each item in the instrument.

The Compassion Fatigue Scale EFA indicated that there was one dominant factor with high item loadings of between 0.541 and 0.873, which is expected given the unidimensional structure of the ProQOL. The Kaiser–Meyer–Olkin (KMO) statistic of sampling adequacy was 0.812, showing that the data were appropriate to perform a factor analysis. EFA of the Organization Performance Questionnaire indicated a single‐factor structure with the same loadings of 0.612 to 0.891; KMO = 0.903. EFA of the Nurses Wellbeing Scale showed a two‐factor solution that matched the physical/behavioral and psychological/relational wellbeing domains of the instrument with loadings (0.538 to 0.862); KMO = 0.881.

Structural equation modeling in AMOS 23.0 was used to conduct CFA by testing how the EFA‐derived factor structures fit the data. The CFA ensured proper model fit in all three instruments (Table 2). The Compassion Fatigue Scale, Organization Performance Questionnaire, and Nurses Wellbeing Scale had significant factor loadings (*p* < 0.001) of 0.54 to 0.91, 0.61 to 0.89, and 0.57 to 0.88, respectively. Such findings imply that the constructs are clear and that the items translated accurately reflect the latent variables they are supposed to represent.

**TABLE 2:** Reliability statistics and CFA fit indices for study instruments.

Instrument	Items	Item‐total *r* range	Cronbach’s *α*	CFA CFI	CFA IFI	RMSEA
Compassion Fatigue (ProQOL STS subscale)	10	0.541–0.873	0.868	0.951	0.953	0.058
Organization Performance Questionnaire	11	0.612–0.891	0.944	0.964	0.966	0.049
Nurses’ Wellbeing Scale	17	0.538–0.862	0.945	0.942	0.944	0.061

*Note:* Acceptable fit: CFI ≥ 0.90; RMSEA ≤ 0.08.

Abbreviations: CFI, comparative fit index; IFI, incremental fit index; RMSEA, root mean square error of approximation.

#### 2.8.4. Corrected Item‐Total Correlations

The item‐total correlations of the Compassion Fatigue Scale were found to be corrected, with all items being positively and significantly correlated with the total scale score (range: 0.541 to 0.873), which indicates that all items serve the purpose of the construct. Organization Performance Questionnaire corrected item‐total correlations of the questionnaire were between 0.612 and 0.891, which indicates a strong homogeneity. The item‐total correlations of the Nurses Wellbeing Scale were between 0.538 and 0.862. These findings substantiate that everything is positively and significantly correlated with the respective scales and is measuring the same underlying constructs.

#### 2.8.5. Internal Consistency

The internal consistency of all of the instruments was determined using Cronbach’s alpha. The score of values was between good (0.868 of the Compassion Fatigue Scale) and excellent (0.944 and 0.945 of perceived organizational performance and wellbeing of nurses, respectively), which validates the acceptable‐to‐excellent reliability of all the tools and facilitates the application of the tools to the Egyptian critical care nursing context.

### 2.9. Ethical Considerations

The Research Ethics Committee of the Faculty of Nursing, Alexandria University granted ethical approval (IRB registration number: IRB00013620; approval date: March 14, 2023, before the start of data collection in July 2024). The AMUH administrative authorities had been informed, and subsequently written institutional approval was obtained. All participants gave informed written consent before receiving questionnaires. The participants were made well aware of their right to withdraw without any penalty at any time. Data collection and storage were done anonymously; no personal identifiers were recorded, and confidentiality was observed during the study.

### 2.10. Data Collection

The questionnaires were printed and manually distributed to qualified nurses in all participating units and working shifts between July 2024 and September 2024 (a 3‐month data collection period). The participants were left to fill out the questionnaire by themselves in a guarded time, which was around 15–20 min. The forms that were filled in were sent to the research team through sealed envelopes to maintain anonymity and minimize bias in response.

### 2.11. Data Analysis

IBM SPSS Statistics Version 23.0 and IBM AMOS Version 23.0 were used to analyze the data. The sociodemographic variables were described using frequencies and percentages, and continuous variables using means and standard deviations (SDs). Pearson correlation coefficient (*r*) was used to evaluate bivariate relationships between the three variables of the study. The mediation model was tested with path analysis by the PROCESS macro (Hayes, Model 4, 5000 bootstrap samples), and the significance of the indirect effect was identified by a bias‐corrected 95% confidence interval. All analyses were established at a level of statistical significance of *p* ≤ 0.05. IBM SPSS Statistics 23.0 and IBM AMOS 23.0 were the versions available under the institutional software license at the time of data collection and analysis (2024–2025); the authors confirm that no newer licensed versions were accessible to the research team during the study period. As a supporting sensitivity analysis (Section 3.2), one‐way ANOVA and Kruskal–Wallis tests, Welch’s *t*‐tests, partial correlations, a covariate‐adjusted ordinary least squares regression model, and interaction (moderation) terms were used to test whether years of nursing experience and unit assignment (ICU vs. non‐ICU) confounded or moderated the study pathway (see Supporting files: *Syntax1.sps to Syntax15.sps, Output CF.spv, Data CF.sav, AmosP1 to AmosP3*).

## 3. Results

As shown in Table 3, the 111 participants were mostly young females, 69.4% of whom were in the age group of 20–29 years (mean age = 27.58 ± 4.62 years) and 64.9% were female. Most of them were single (59.5%), and they had a bachelor’s degree in nursing (78.4%). The majority of them worked in the ICU (54.1%), followed by the ER and outpatient clinics (13.5% each). Nursing experience was relatively young (48.6% with less than 5 years of general experience) and early‐career oriented (64.0% with less than 5 years of current‐unit experience), which is why the sample predominantly consisted of young nurses with minimal years of experience.

**TABLE 3:** Participants’ distribution according to personal and work‐related characteristics (*n* = 111).

Demographic and professional data	No.	%
Gender		
Male	39	35.1
Female	72	64.9
Age (years)		
20–< 30	77	69.4
30–< 40	31	27.9
40–< 50	3	2.7
Mean ± SD	27.58 ± 4.62	
Marital status		
Single	66	59.5
Married	44	39.6
Divorced	1	0.9
Working Unit		
ICU	60	54.1
ER	15	13.5
Clinics	15	13.5
Employee and supervisory	5	4.5
Specialty units (Dialysis, Cardio, Abdominal, Liver, Pediatric, Geriatric, Community, Hematemesis)	16	14.4
Qualification		
Bachelor’s degree in nursing	87	78.4
Two‐year postsecondary nursing diploma	12	10.8
Master’s degree in nursing	11	9.9
High school nursing diploma	1	0.9
Years of nursing experience (General)		
< 5 years	54	48.6
5–10 years	37	33.3
11–20 years	16	14.4
> 20 years	4	3.6
Mean ± SD	7.50 ± 5.33	
Years of experience in current unit		
< 5 years	71	64.0
5–10 years	29	26.1
11–20 years	9	8.1
> 20 years	2	1.8
Mean ± SD	5.36 ± 4.61	

According to Table 4, most participants (65.8%) had low overall compassion fatigue (mean = 2.70 ± 0.74) and 27.0% moderate fatigue. Preoccupation with the people being helped (Item 1: mean = 3.68 ± 0.80; 55.0% high) was the highest rated item, which showed that intrusive preoccupation is noted to be widespread in the early stages, even among low overall fatigue nurses. Perceived traumatic stress contagion (Item 4: mean = 3.09 ± 1.08; 36.0% high) and depression based on patient experiences (Item 6: mean = 2.92 ± 1.24; 32.4% high) were also significantly supported. The least common were avoidance behaviors and intrusive frightening thoughts (Items 8 and 9), which implies that the extreme sequelae of compassion fatigue is still relatively kept in check, whereas intrusive emotional concern is already common in the early stage. It should be noted that the 65.8% figure reflects the proportion of nurses individually classified as having “low” compassion fatigue using the < 50%‐of‐score‐range cutoff (individual mean score < 3.0) applied at the person level; the overall sample mean of 2.70 corresponds to 42.5% of the possible score range ((2.70 − 1)/4 × 100), which is consistent with most nurses falling in the low category.

**TABLE 4:** Participants’ levels of perception regarding compassion fatigue (*n* = 111).

Items	Mean	±	SD	Low no.	Low %	Moderate no.	Moderate %	High no. (%)
1. I am preoccupied with more than one person I help.	3.68	±	0.80	4	3.6	46	41.4	61 (55.0%)
2. I jump or am startled by unexpected sounds.	2.68	±	1.02	48	43.2	40	36.0	23 (20.7%)
3. I find it difficult to separate my personal life from my role as a helper.	2.67	±	1.15	48	43.2	39	35.1	24 (21.6%)
4. I think I may have been affected by the traumatic stress of those I help.	3.09	±	1.08	30	27.0	41	36.9	40 (36.0%)
5. Because of my helping, I have felt “on edge” about various things.	2.77	±	1.08	41	36.9	45	40.5	25 (22.5%)
6. I feel depressed because of the traumatic experiences of the people I help.	2.92	±	1.24	39	35.1	36	32.4	36 (32.4%)
7. I feel as though I am experiencing the trauma of someone I have helped.	2.36	±	1.01	64	57.7	32	28.8	15 (13.5%)
8. I avoid situations that remind me of frightening experiences of the people I help.	2.32	±	1.20	72	64.9	18	16.2	21 (18.9%)
9. As a result of my helping, I have intrusive, frightening thoughts.	2.15	±	1.16	72	64.9	24	21.6	15 (13.5%)
10. I cannot recall important parts of my work with trauma victims.	2.32	±	1.13	68	61.3	25	22.5	18 (16.2%)
Overall compassion fatigue	2.70	±	0.74	73	65.8%	30	27.0%	8 (7.2%)

*Note:* Low, < 50% of the possible score range (mean < 3.0); moderate, 50% to < 75% (mean 3.0 to < 4.0); high, ≥ 75% (mean ≥ 4.0), where percentage (mean − 1)/4 × 100. Scores based on five‐point Likert scale.

As shown in Table 5, 44.1% of respondents perceived organizational performance as low (mean = 3.04 ± 0.84) and the remaining 40.5% perceived it as moderate. Succession readiness (Item 10: mean = 3.57 ± 1.06; 61.3% high) was rated the best, whereas career growth policies (Item 8: mean = 2.89 ± 1.05), adaptation to operational change (Item 3), and equitable performance appraisal (Items 4 and 7) were rated the lowest. These results indicate that the workforce sees its institutions as working well but poorly developed in terms of strategic development. Notably, Item 10 (“If you left your job tomorrow, someone in your unit could immediately take over”), although scored as the most positive item, may also be interpreted by some respondents as reflecting low perceived personal indispensability rather than purely organizational succession strength; this potential ambiguity in construct interpretation is noted for readers.

**TABLE 5:** Participants’ levels of perception regarding perceived organization performance (*n* = 111).

Items	Mean	±	SD	Low no.	Low %	Moderate no.	Moderate %	High no. (%)
1. My organization practices effective two‐way communication.	3.11	±	1.05	34	30.6	34	30.6	43 (38.7%)
2. My organization has a clear sense of direction and focus.	3.02	±	1.08	37	33.3	37	33.3	37 (33.3%)
3. My organization rapidly adapts to needed operational changes.	2.92	±	1.07	42	37.8	38	34.2	31 (27.9%)
4. My organization practices effective planning at all levels.	2.95	±	1.00	38	34.2	42	37.8	31 (27.9%)
5. My organization places a high priority on workforce training and development.	3.13	±	1.03	31	27.9	38	34.2	42 (37.8%)
6. My organization conducts formal performance appraisals regularly.	3.05	±	1.09	39	35.1	29	26.1	43 (38.7%)
7. My performance on the job is evaluated fairly.	2.95	±	1.09	42	37.8	34	30.6	35 (31.5%)
8. My organization has policies that encourage career growth and development.	2.89	±	1.05	42	37.8	37	33.3	32 (28.8%)
9. My organization builds a deep reservoir of successors at every level.	2.96	±	0.96	38	34.2	42	37.8	31 (27.9%)
10. If you left your job tomorrow, someone in your unit could immediately take over.	3.57	±	1.06	16	14.4	27	24.3	68 (61.3%)
11. My organization has policies that encourage professional development opportunities.	2.93	±	1.05	38	34.2	40	36.0	33 (29.7%)
Overall perceived organization performance	3.04	±	0.84	49	44.1%	45	40.5%	17 (15.3%)

*Note:* Low, mean < 3.0 (unsatisfied). Moderate and high, mean ≥ 3.0 (satisfied). Classification based on instrument scoring criteria.

The results of the nurses’ wellbeing are given in Table 1. The overall mean wellbeing score was 4.02 ± 1.98 (range of the 1–10 item scale), which is below the scale midpoint of 5.5; 75.7% of nurses were in the low category, 19.8% in the moderate category, and 4.5% in the high category. Items 2–5 (negatively worded) are shown after reverse scoring (11 − raw score) so that higher values indicate higher wellbeing on every item. Career meaningfulness (Item 1: mean = 5.07 ± 3.14) was the highest rated item, followed by Item 3 (coworkers’ work‐related burnout; reverse‐scored mean = 4.98 ± 2.94). All reverse‐scored items had means below the scale midpoint (Item 2: 4.05 ± 2.50; Item 3: 4.98 ± 2.94; Item 4: 3.91 ± 2.71; Item 5: 4.69 ± 3.02), indicating that nurses perceived high levels of stress and burnout among their coworkers and a substantial negative effect of COVID‐19 and work strain on their wellbeing. In contrast, physical self‐care behaviors showed the lowest ratings: exercise (Item 12: mean = 2.77 ± 2.47; 87.4% low), healthy sleep habits (Item 13: mean = 2.89 ± 2.48; 85.6% low), and mindfulness practices (Item 15: mean = 2.96 ± 2.51; 83.8% low). Comfort in discussing wellbeing with managers was also low (Item 14: mean = 3.14 ± 2.58; 80.2% low), which points to limited psychological safety in the nurse–manager relationship.

Table 6 presents the results of the correlations among compassion fatigue, perceived organizational performance, and nurses’ wellbeing. The findings revealed that compassion fatigue was significantly and negatively correlated with both perceived organizational performance (*r* = −0.400, *p* < 0.001) and nurses’ wellbeing (*r* = −0.513, *p* < 0.001). Nurses’ wellbeing was significantly and positively correlated with perceived organizational performance (*r* = 0.619, *p* < 0.001). The bold values in the correlation matrix represent the focus of the highest significant results and support findings H1, H2, and H3.

**TABLE 6:** Correlations between compassion fatigue, perceived organization performance, and nurses’ wellbeing (*n* = 111).

Variable	Compassion fatigue	Perceived organization performance	Nurses’ wellbeing
Compassion fatigue			
Perceived organization performance	*r* = −0.400^∗^ *p* < 0.001^∗^		
Nurses’ wellbeing	*r* = −0.513^∗^ *p* < 0.001^∗^	*r* = 0.619^∗^ *p* < 0.001^∗^	

*Note: r*, Pearson correlation coefficient. Correlations are based on composite scores (*n* = 111).

^∗^Statistically significant at *p* ≤ 0.05.

### 3.1. Path Analysis and Mediation Results

The path analysis and mediation findings are presented in Table 7; the path diagram with unstandardized coefficients is shown in Figure 2. Compassion fatigue significantly predicted nurses’ wellbeing (path a: *B* = −2.329, SE = 0.374, *t* = −6.23, *p* < 0.001; *R* ^2^ = 0.263), supporting H2; compassion fatigue explained 26.3% of the variance in nurses’ wellbeing. Nurses’ wellbeing was a significant predictor of perceived organizational performance when compassion fatigue was controlled (path b: *B* = 0.154, SE = 0.024, *t* = 6.43, *p* < 0.001; *R* ^2^ of the full model = 0.392), supporting H3. When wellbeing was controlled, the direct effect of compassion fatigue on perceived organizational performance was nonsignificant (direct path c′: *B* = −0.140, SE = 0.109, 95% CI [−0.356, 0.077], *p* = 0.203; 28.0% of the total effect). The indirect effect of compassion fatigue on perceived organizational performance through wellbeing was significant (*B* = −0.360, bootstrap SE = 0.081, 95% bias‐corrected bootstrap CI [−0.545, −0.227]; 72.0% of the total effect), supporting H4 (statistical mediation). Direct and indirect effect proportions sum to 100% of the total effect. The total effect of compassion fatigue on perceived organizational performance was significant (total effect: *B* = −0.500, SE = 0.110, 95% CI [−0.717, −0.282], *t* = −4.55, *p* < 0.001). Because the data are cross‐sectional, these results describe statistical mediation and should not be read as proof of a causal sequence.

**TABLE 7:** Path analysis: mediating role of nurses’ wellbeing in the relationship between compassion fatigue and perceived organization performance (*n* = 111).

Dependent variable		Independent variable	*B*	*R* ^2^	SE	95% CI LL	95% CI UL	*t*	*p* value	Effect proportion (%)
Model paths	Nurses’ wellbeing	Compassion fatigue	−2.329	0.263	0.374	−3.070	−1.588	−6.23^∗^	< 0.001^∗^	—
	Perceived Org. performance	Nurses’ wellbeing	0.154	0.392	0.024	0.107	0.202	6.43^∗^	< 0.001^∗^	—

Type of effect	Direct effect		−0.140	—	0.109	−0.356	0.077	−1.28	0.203	28.0
	Indirect effect		−0.360	—	0.081	−0.545	−0.227	—	Sig.^∗^	72.0
	Total effect		−0.500	—	0.110	−0.717	−0.282	−4.55^∗^	< 0.001^∗^	—

*Note: B*, unstandardized regression coefficient. Bootstrap samples = 5000; the CI for the indirect effect is a bias‐corrected bootstrap CI and its SE is the bootstrap SE. *R* ^2^ in the first row is for the model predicting wellbeing from compassion fatigue; *R* ^2^ in the second row is for the model predicting perceived organizational performance from compassion fatigue and wellbeing. Total effect *R* ^2^ = 0.160. Estimates were computed from summed scale scores.

Abbreviations: CI, confidence interval; LL, lower limit; SE, standard error; UL, upper limit.

^∗^Statistically significant at *p* ≤ 0.05.

**FIGURE 2:**
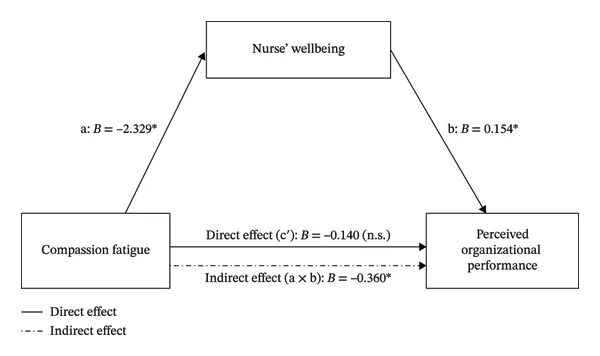
Path diagram with unstandardized path coefficients *B*; ^∗^ *p* < 0.05 or bootstrap CI excluding zero.

### 3.2. Sensitivity Analysis: Robustness of the Pathway to Nursing Experience and Unit Assignment

In response to a reviewer request to test whether the demographic and occupational characteristics of the sample confound or moderate the study pathway, given that 48.6% of nurses had less than five years of experience, four supporting checks were conducted using years of nursing experience (grouped as < 5, 5–10, 11–20, and > 20 years) and unit assignment (ICU, including general and specialty ICU, versus non‐ICU units comprising the ER, outpatient, and other administrative/support units): (1) one‐way ANOVA and Kruskal–Wallis tests comparing compassion fatigue, wellbeing, and perceived organizational performance across experience groups and between ICU and non‐ICU nurses; (2) partial correlations between the three study variables controlling for experience and unit; (3) a covariate‐adjusted sensitivity regression model, in which experience and unit were entered as covariates alongside the study paths, compared against the corresponding unadjusted estimates; and (4) interaction (moderation) terms testing whether experience or unit altered the strength of the compassion fatigue ⟶ wellbeing and compassion fatigue ⟶ perceived organizational performance associations. Results are summarized in Table 8 (see Supporting files: *Syntax1.sps to Syntax15.sps, Output CF.spv, Data CF.sav, AmosP1 to AmosP3*).

**TABLE 8:** Sensitivity analysis: compassion fatigue, wellbeing, and perceived organizational performance by nursing experience and unit assignment, partial correlations, covariate‐adjusted model, and moderation tests (*n* = 111).

Analysis/Variable/Path	Value 1	Value 2	Value 3	Value 4	Test statistic	*p* value/interpretation
*Panel A. Subgroup Comparison by Years of Nursing Experience (One-Way ANOVA/Kruskal–Wallis)*						
Compassion Fatigue, M (SD)	< 5 years: 2.63 (0.69)	5–10 years: 2.84 (0.88)	11–20 years: 2.54 (0.52)	> 20 years: 2.93 (0.61)	*F* (3107) = 0.96; *H* = 2.29	*p* = 0.413 (*F*); *p* = 0.515 (*H*)
Wellbeing, M (SD)	< 5 years: 3.78 (1.58)	5–10 years: 4.18 (2.46)	11–20 years: 4.58 (2.06)	> 20 years: 3.57 (1.42)	*F* (3107) = 0.83; *H* = 1.63	*p* = 0.479 (*F*); *p* = 0.653 (*H*)
Perceived Organizational Performance, M (SD)	< 5 years: 2.98 (0.80)	5–10 years: 3.02 (0.88)	11–20 years: 3.38 (0.88)	> 20 years: 2.80 (0.75)	*F* (3107) = 1.06; *H* = 2.46	*p* = 0.369 (*F*); *p* = 0.483 (*H*)

*Panel B. Subgroup Comparison by Unit (ICU vs. Non-ICU) (Welch’s t-test)*						
Compassion Fatigue, M (SD)	ICU: 2.65 (0.73)	Non‐ICU: 2.75 (0.75)	—	—	*t* = −0.73	*p* = 0.468
Wellbeing, M (SD)	ICU: 4.03 (1.97)	Non‐ICU: 4.02 (2.00)	—	—	*t* = 0.03	*p* = 0.975
Perceived Organizational Performance, M (SD)	ICU: 3.09 (0.94)	Non‐ICU: 2.99 (0.71)	—	—	*t* = 0.59	*p* = 0.555

*Panel C. Partial Correlations Controlling for Experience and Unit (vs. Zero-Order)*						
Compassion Fatigue—Wellbeing	Zero‐order *r* = −0.513 (*p* < 0.001)	Partial *r* = −0.518 (*p* < 0.001)	—	—	—	Sig. in both
Wellbeing—Perceived Organizational Performance	Zero‐order *r* = 0.619 (*p* < 0.001)	Partial *r* = 0.614 (*p* < 0.001)	—	—	—	Sig. in both
Compassion Fatigue—Perceived Organizational Performance	Zero‐order *r* = −0.400 (*p* < 0.001)	Partial *r* = −0.402 (*p* < 0.001)	—	—	—	Sig. in both

*Panel D. Covariate-Adjusted Sensitivity Regression Model (Unadjusted vs. Adjusted for Experience + Unit)* ^a^						
Path a: Compassion Fatigue ⟶ Wellbeing	Unadjusted *B* = −2.329 (*p* < 0.001)	Adjusted *B* = −2.347 (*p* < 0.001)	—	—	—	Sig. in both
Path b: Wellbeing ⟶ Perceived Org. Performance (controlling CF)	Unadjusted *B* = 0.154 (*p* < 0.001)	Adjusted *B* = 0.152 (*p* < 0.001)	—	—	—	Sig. in both
Path c’: Compassion Fatigue ⟶ Perceived Org. Performance (direct, controlling Wellbeing)	Unadjusted *B* = −0.140 (*p* = 0.203)	Adjusted *B* = −0.142 (*p* = 0.202)	—	—	—	n.s. in both
Indirect effect (*a* × *b*), 95% bootstrap CI	Unadjusted: −0.360 (−0.545, −0.227)	Adjusted: −0.358 (−0.545, −0.222)	—	—	—	Sig. in both

*Panel E. Moderation (Interaction) Tests*						
Compassion Fatigue × Experience ⟶ Wellbeing	*B* = −0.161	—	—	—	—	*p* = 0.764
Compassion Fatigue × Experience ⟶ Perceived Org. Performance	*B* = −0.224	—	—	—	—	*p* = 0.153
Compassion Fatigue × Unit ⟶ Wellbeing	*B* = −0.403	—	—	—	—	*p* = 0.595
Compassion Fatigue × Unit ⟶ Perceived Org. Performance	*B* = −0.040	—	—	—	—	*p* = 0.858

*Note:* ICU, intensive care unit (general and specialty ICU). Non‐ICU, emergency room, outpatient, and other administrative/support units. *F*, one‐way ANOVA statistic. *H*, Kruskal–Wallis statistic. *t*, Welch’s *t*‐test statistic. *B*, unstandardized regression coefficient. Panels A and B report composite means on the original item scales (*compassion fatigue and perceived organizational performance*, 1–5; *wellbeing*, 1–10); Panels C–E use summed scores as in Tables 6 and 7. Interaction terms were tested with mean‐centered compassion fatigue; experience was coded 1–4 and unit 1 = ICU, 2 = non‐ICU.

^a^Panel D reports a covariate‐adjusted regression‐based (PROCESS Model 4) sensitivity model using summed scale scores, conducted as a robustness check on the direction and significance of the pathway; its unadjusted column reproduces the primary mediation model reported in Table 7.

Abbreviations: M (SD), mean (standard deviation); n.s., not significant.

Neither compassion fatigue, wellbeing, nor perceived organizational performance differed significantly across the four experience groups (all *p* ≥ 0.369) or between ICU and non‐ICU nurses (all *p* ≥ 0.468) (Table 8, Panels A and B). Partial correlations controlling for experience and unit were virtually identical to the zero‐order coefficients (differences ≤ 0.005) and did not alter the significance pattern of any relationship (Table 8, Panel C). In the covariate‐adjusted sensitivity regression model, the path estimates were essentially unchanged: the direct compassion fatigue ⟶ perceived organizational performance path remained nonsignificant (unadjusted *B* = −0.140, *p* = 0.203; adjusted *B* = −0.142, *p* = 0.202), the compassion fatigue ⟶ wellbeing path remained significant (unadjusted *B* = −2.329; adjusted *B* = −2.347; both *p* < 0.001), the wellbeing ⟶ perceived organizational performance path remained significant (unadjusted *B* = 0.154; adjusted *B* = 0.152; both *p* < 0.001), and the indirect effect remained significant (unadjusted *B* = −0.360, 95% CI [−0.545, −0.227]; adjusted *B* = −0.358, 95% CI [−0.545, −0.222]) (Table 8, Panel D). Taken together, these findings indicate that the compassion fatigue–wellbeing–perceived organizational performance pathway reported in Table 7 is not attributable to, and is not confounded or moderated by, nurses’ years of experience or unit assignment (see Supporting files: *Syntax1.sps to Syntax15.sps, Output CF.spv, Data CF.sav, AmosP1 to AmosP3*).

## 4. Discussion

Nursing departments need ways to protect nurses and to sustain the organizational functioning on which nursing safety depends. This study examined whether nurses’ wellbeing mediates the association between compassion fatigue (measured as STS) and perceived organizational performance among nurses at AMUH, Alexandria, Egypt. The hypothesized mediation (H4) was supported statistically: wellbeing accounted for most of the association between compassion fatigue and perceived organizational performance. The study also identified wellbeing deficits relevant to nursing management, especially in physical self‐care and manager–nurse communication. Because the design is cross‐sectional, these findings are associations and do not establish causal order.

### 4.1. Compassion Fatigue Levels and Perceived Organizational Performance

In our sample, most nurses (65.8%) had low overall compassion fatigue levels (mean = 2.70 ± 0.74), while 7.2% had high compassion fatigue levels. H1 was supported: Compassion fatigue was significantly and negatively correlated with perceived organizational performance (*r* = −0.400, *p* < 0.001). This suggests that even low‐to‐moderate levels of compassion fatigue (STS) are associated with less favorable perceptions of perceived organizational performance, so clinically significant levels may not be required for such an association to appear. Because the study is cross‐sectional and self‐reported, the direction of this association cannot be established; nurses who view their organization less favorably may also report more distress, and shared influences such as workload may contribute to both.

One possible explanation draws on COR theory [13], which holds that even partial resource loss reduces the effort people can direct toward goals beyond immediate demands. Nurses experiencing early compassion fatigue may therefore have fewer resources to engage with organizational activities and may rate organizational functioning less favorably, although the present data cannot test this mechanism. Our finding is consistent with earlier studies that linked compassion fatigue and occupational stress with poorer organizational and work outcomes in acute care settings [18, 19].

Also worth mentioning, the findings for our moderate‐level compassion fatigue reflect current workforce facts in the whole nation. With majority young and beginning nurse profession in ICU environment in Egypt, mean of experience less than 5 years for 48.6% of nurses, they might not still reach high compassion fatigue level in comparison to nurses with longer experience, yet early stage of intrusive preoccupation shown in our finding and more clearly Item 1 (55.0%) in the intrusion section might indicate the early beginning and moving toward severe level. This finding concurs with Cocker and Joss [3] and Kelly et al. [18] caution not to consider the early‐career nurse population at no risk.

### 4.2. Nurses’ Wellbeing Analysis Findings

The most pronounced wellbeing deficits among the nurses in our sample lay in physical self‐care behaviors and organizational communication about wellbeing, whereas work meaning and career fulfillment were comparatively higher, although still below the scale midpoint. Overall wellbeing was low (mean = 4.02 ± 1.98 on the 1–10 scale). H2 was supported: Compassion fatigue was significantly and negatively associated with nurses’ wellbeing (*B* = −2.329, *R* ^2^ = 0.263, *p* < 0.001), meaning that compassion fatigue accounted for 26.3% of the variance in wellbeing. This is consistent with the resource‐loss process described by COR theory [13], although the cross‐sectional data cannot confirm that compassion fatigue leads to lower wellbeing rather than the reverse.

One possible reason for these physical self‐care deficiencies, although working hours were not measured in this study, is the reality of Egyptian ICU nurses’ working schedules, in which their shifts extend significantly past normal hours with minimal time for recovery, precluding them from partaking in the exercise, sufficient sleep, and mindfulness that are crucial for the fulfillment of wellbeing needs. This finding corroborates Dall’Ora et al. [19] and West et al. [20], who found shift length and recovery time to be among the most predictive structural determinants of nurse wellbeing within the medical system. The other possible reason is that our finding that 80.2% of nurses feel uncomfortable discussing wellbeing issues with their managers suggests that the institutional culture does not make psychological safety available for nurses to seek help and support for their wellbeing from their superior managers, which supports El‐Sayed et al. [21] in that collaborative and knowledge‐sharing leadership practices provide nurses a sense of ownership and involvement within the working system, unlike Keener [7] and HealthStream [12], in which lack of manager receptivity to discussing wellbeing with their staff is among the strongest predictors of nurse support‐seeking.

### 4.3. The Mediating Role of Nurses’ Wellbeing

Our results indicate that nurses’ wellbeing statistically mediated the association between compassion fatigue and perceived organizational performance (indirect effect *B* = −0.360, 95% bootstrap CI [−0.545, −0.227]; 72.0% of the total effect), so H4 was supported. The direct effect became nonsignificant once wellbeing was included (*p* = 0.203; 28.0% of the total effect). This pattern is compatible with wellbeing being one pathway through which compassion fatigue and perceived organizational performance are linked, and it remained unchanged after adjusting for experience and unit (Table 8). However, mediation inferred from cross‐sectional data cannot show temporal order, and alternative models (for example, wellbeing influencing compassion fatigue, or a third variable influencing all three) cannot be excluded.

One possible explanation is that compassion fatigue may not directly reduce work engagement but may draw down the wellbeing resources (physical energy, emotional robustness, and self‐regulation) that nurses need to take part in work and patient care. When wellbeing resources are sufficient, the costs of compassion fatigue may be borne without disengagement, whereas when they are depleted, withdrawal is a plausible response. This interpretation is congruent with the logic of the JD‐R model [14] and with mediation studies in neighboring fields [22, 23], but it remains to be tested with longitudinal data.

In line with our result, Maslach and Leiter [24] described how occupational stress and burnout are linked with lower performance through depletion of engagement resources. Related mediation studies in nursing have mostly examined compassion fatigue as the mediator or outcome: Liu X et al. [25] found that compassion fatigue partially mediated the relationship between perceived organizational support and caring behavior among outpatient nurses in China, and Liu D et al. [26] found that perceived organizational support partially mediated the relationship between occupational stressors and compassion fatigue among frontline nurses. The specific contribution of the present study is that, to our knowledge, it is the first in an Egyptian hospital setting to test nurses’ wellbeing as an intervening variable between compassion fatigue and perceived organizational performance using bootstrapped path analysis, and that it documents specific wellbeing deficits (exercise, sleep, mindfulness, and comfort discussing wellbeing with managers) together with psychometric data for Arabic versions of the three instruments. These findings suggest that organizational interventions that support nurse wellbeing deserve testing as ways to weaken the compassion fatigue–performance association.

## 5. Conclusion

This research examined nurses’ wellbeing as a mediating factor in the link between compassion fatigue (measured as STS) and perceived organizational performance among nurses at an Egyptian university hospital. Nurses’ wellbeing statistically mediated this association: compassion fatigue explained 26.3% of the variance in nurses’ wellbeing, and the indirect path through wellbeing accounted for 72.0% of the total association between compassion fatigue and perceived organizational performance. The analysis further revealed two domains in which wellbeing was most deficient and where the organization has the most potential to act: physical self‐care behavior and managers’ communication about nurses’ wellbeing. Within the limits of a cross‐sectional, single‐site, self‐reported design, nursing leaders may consider nurses’ wellbeing as an important organizational priority for sustaining perceived organizational performance.

### 5.1. Implications for Nursing Management

These results suggest several areas in which nursing management and leadership may target resources; because of the cross‐sectional design, these recommendations are suggestions to be tested rather than proven causal pathways. Integrating screening for routine compassion fatigue into workforce management using standardized measures like the ProQOL is needed to provide early identification and intervention before health is severely impacted. Deficits in sleep, exercise, and mindfulness were widespread and may partly reflect organizational factors (e.g., shift patterns and workload) rather than only individual choices, and may therefore be addressed organizationally with things like protecting schedules, providing breaks, and workplace resources. Fostering safe environments where nurses feel they can speak about their wellbeing to their supervisor is also imperative and requires leadership education in supportive communication, active listening, and compassionate management. Creating systems for peer support, like peer mentoring, structured debriefs, and interdisciplinary mental support, needs to be built into standard practice in the ICU. Nursing education curriculum needs to be expanded to focus on health and prevention of compassion fatigue. Last, hiring and developing nurse leaders who practice in supportive and collaborative ways needs to be prioritized.

### 5.2. Policy Implications

At the institutional level, AMUH and other public hospitals ought to incorporate formal nurse wellbeing monitoring programs within quality governance frameworks that include clearly established protocols and concrete procedures when wellbeing issues are raised. Incorporating the indicators into daily processes would ensure that nurses’ wellbeing is considered as a crucial part of the institution’s performance indicators, rather than a discretionary issue. At the same time, national accreditation institutions ought to make compassion fatigue screening and wellbeing indicators part of the evaluation standards for hospitals, thus establishing mechanisms for accountability and ensuring continuous efforts toward staff wellbeing development at the institutional level.

At the national level, there needs to be well‐defined and evidence‐based policies that govern nurse–patient staffing ratios and shift hours so as to ensure that the time required for recovery and physical wellbeing is guaranteed. Dedicated allocations of health resources within each country’s health sector for occupational health support programs (including management of compassion fatigue, peer support systems, and resilience development) need to be made sustainable. Accessibility to professionally developed training and resource materials dealing with psychological wellbeing and health at work needs to be increased for nurses. The significance of these findings at the regional level spans Middle Eastern and North African healthcare systems where similar constraints toward workforce and resources may be seen. Establishing unified regional criteria for measuring nurse wellbeing and compassion fatigue could strengthen and standardize nursing workforces in the region, and contribute to healthier nursing work environments.

### 5.3. Research Strengths and Limitations

This paper provides new and relevant data on the organizational outcomes of compassion fatigue among Egyptian critical care nurses. Furthermore, to the best of the authors’ knowledge, it is the first to investigate the wellbeing‐mediated pathway from compassion fatigue to perceived organizational performance through a path analysis with bootstrapping methodology within a hospital in Egypt. It contributes to the nursing literature by testing a wellbeing‐mediated pathway using a composite wellbeing score and by identifying specific wellbeing deficits (physical self‐care and manager communication) that can inform targeted interventions. Our study utilized three instruments with high reliability coefficients and tested three validated measures psychometrically utilizing EFA, CFA, and corrected item‐total correlations adapted for an Egyptian sample. Bootstrapped path analysis (5000 resamples) is a robust approach for testing indirect effects.

There are certain limitations that need to be acknowledged. The cross‐sectional nature of the design limits the ability to draw causal inferences, despite the theoretical foundation and statistical significance of the mediation model. Further longitudinal investigation is needed to support temporal ordering of the variables. The use of self‐report instruments may introduce social desirability bias, especially for items related to self‐care and manager relationship. Limitations are related to the sampling strategy of the present study, as convenience sampling from a single site cannot be used to generalize results to other governorates of Egypt, private hospitals, or other international settings. Further studies are needed to use longitudinal designs, recruit from multiple hospitals including private and regional, and include objective measures of performance. Compassion fatigue was operationalized using only the ProQOL STS subscale; the burnout and compassion satisfaction subscales were not administered, so the findings may not generalize to the full compassion fatigue construct. Perceived organizational performance was measured through nurses’ self‐reported perceptions rather than objective institutional indicators. Only 54.1% of participants worked in intensive care and 13.5% in the ER; the remainder worked in other inpatient and outpatient units, so the findings should not be generalized specifically to critical care nurses. Given the sample size (*n* = 111) relative to the number of items in the three confirmatory factor models, particularly the two‐factor, 17‐item Nurses’ Wellbeing Scale, the parameter‐to‐sample ratio was below some conventional recommendations (e.g., 10:1 or a minimum of approximately 200) for stable CFA estimation; this is acknowledged as a further limitation, and replication with a larger sample is recommended. Because compassion fatigue, wellbeing, and perceived organizational performance were all measured by self‐report from the same respondents at the same time point, common method variance may have inflated the observed associations; this limitation, beyond the general cross‐sectional caveat above, should be considered when interpreting the strength of the reported relationships, and future studies should incorporate procedural or statistical remedies (e.g., temporally or source‐separated measurement, Harman’s single‐factor test, or a marker‐variable approach). Two of the three instruments used, the Organizational Performance Questionnaire [6] and the Nurses’ Wellbeing Scale [7], originate from unpublished graduate theses rather than peer‐reviewed published instruments; although the present study conducted independent EFA, CFA, and reliability testing and obtained developer permission to use and adapt both tools, the extent to which their psychometric properties have been independently replicated elsewhere remains limited, and this should be taken into account when interpreting findings based on these measures.

## Author Contributions

All authors participated in the research idea, conceptualization, data collection, analysis, and preparation of the manuscript for publication. Sally Mohammed Farghaly Abdelaliem: conceptualization, methodology, writing–original draft and review and editing the final draft, and supervision. Haitham Mokhtar Mohamed Abdallah: formal analysis, data collection, and writing–review and editing. Mohamed Ezzelregal Abdelgawad: validation, data curation, and writing–review and editing.

## Funding

This research received no specific grant from any funding agency in the public, commercial, or not‐for‐profit sectors.

## Ethics Statement

The current study was approved by the Research Ethics Committee of the Faculty of Nursing, Alexandria University (IRB registration number: IRB00013620; approval date: March 14, 2023, before the start of data collection in July 2024). The administrative authority of AMUH gave written permission to conduct the study. All methods were carried out in accordance with relevant guidelines and regulations.

## Consent

All participants gave informed written consent before receiving questionnaires. The participants were made well aware of their right to withdraw without any penalty at any time. Data collection and storage were done anonymously, and no personal identifiers were recorded; confidentiality was observed during the study.

## Conflicts of Interest

The authors declare no conflicts of interest.

## Supporting Information

Additional supporting information can be found online in the Supporting Information section.

## Supporting information


**Supporting Information** *Syntax1.sps to Syntax15.sps, Output CF.spv, Data CF.sav, AmosP1 to AmosP3*.

## Data Availability

The data and materials of the current study are not publicly available due to confidentiality reasons but are available from the corresponding author on reasonable request. The de‐identified SPSS dataset and AMOS/PROCESS syntax used to generate the results in this manuscript are available as supporting information from the corresponding author upon reasonable request, to support reproducibility.
